# Supplementary material for: Bacterial Lipopeptides Are Effective against Pear Fire Blight
Source: Microorganisms. 2024 Apr 29;12(5):896. doi: 10.3390/microorganisms12050896 (PMC11123750; doi:10.3390/microorganisms12050896)
Supplement: Supplementary file 1 [file microorganisms-12-00896-s001.zip › microorganisms-2986285-supplementary.pdf]

Supplementary Information

Table S1. primers used to detect gene for biological control in *B. subtilis* and *P. megaterium*

S/No	Gene	Primer name	Primer sequence (5'-3')	Annealing Temp	Size (bp)	Reference
1	*Spas*	Spas-F<br>Spas-R	GGTTTGTTGGATGGAGCTGT<br>GCAAGGAGTCAGAGCAAGGT	58 °C	375	Song Wenxin (2020)
2	*fenD*	FNDF1<br>FNDR1	CCT GCA GAA GGA GAA GTG AAG<br>TGC TCA TCG TCT TCC GTT TC	52 °C	293	Joshi, R., et al. (2006).
3	*yndJ*	147F<br>147R	CAG AGC GAC AGC AAT CAC AT<br>TGA ATT TCG GTC CGC TTA TC	52 °C	212	Joshi, R., et al. (2006).
4	*srfAA*	SRFAF1<br>SRFAR1	GAA AGA GCG GCT GCT GAA AC<br>CCC AAT ATT GCC GCA ATG AC	62 °C	273	Joshi, R., et al. (2006).
5	*Qk1*	QK1-F<br>QK1-R	CTTAAACGTCAGAGGCGGAG<br>ATTGTGCAGCTGCTTGTACG	55 °C	704	Song Wenxin (2020)
6	*ItuC*	ITUCF1<br>ITUCR3	TTC ACT TTT GAT CTG GCG AT<br>CGT CCG GTA CAT TTT CAC	52 °C	575	Joshi, R., et al. (2006).

Table S2. Chart outlining interpretive standards for zone diameter, used to determine the sensitivity and resistance status of antibiotics through the disk diffusion method [39].

S/no	Name of antibiotics (dose)	Inhibitory zone diameter to nearest millimeter (mm)		
		Sensitive (S)	Moderately sensitive (MS)	Resistant (R)
1	Cefthiopene (30 µg/disk)	≥18mm	15−17 mm	≤14 mm
2	Kanamycin (30 µg/disk)	≥18mm	14−17 mm	≤13 mm
3	Streptomycin (10 µg/disk)	≥15mm	12−14 mm	≤11 mm
4	Erythromycin (15 µg/disk)	≥23mm	14−22 mm	≤13 mm
5	Ciprofloxacin (15 µg/disk)	≥21mm	16−20 mm	≤15 mm
6	Tetracycline (30 µg/disk)	≥15mm	12−14 mm	≤11 mm
7	Penicillin (10 µg/disk)	≥14mm	00−00 mm	≤15 mm
8	Ampicillin (10 µg/disk)	≥13mm	14−16 mm	≤17 mm

Supplementary Information

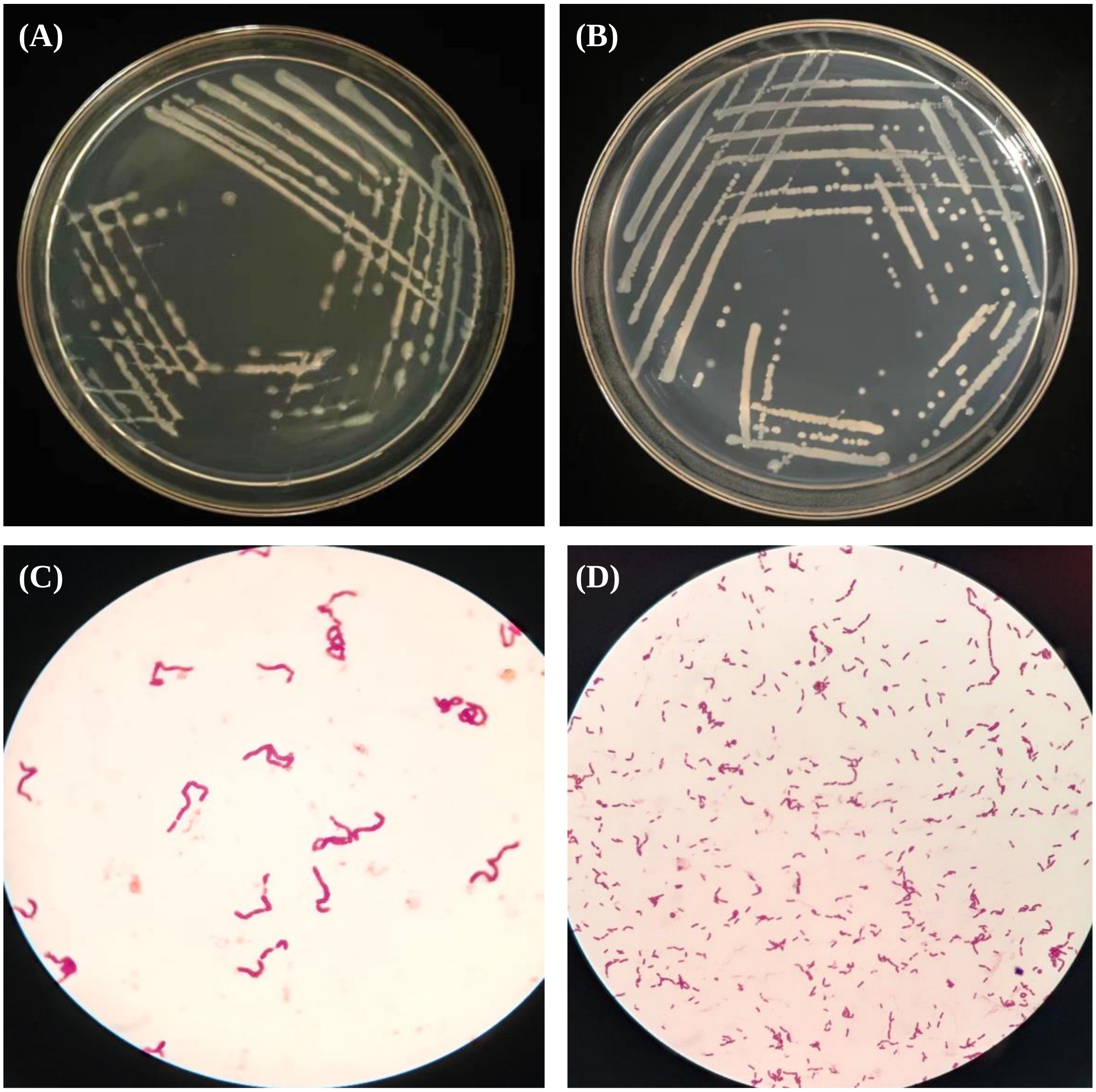

Figure S1. Streaking and pure NB agar plate of Isolate **(A).** *P. megaterium* H1, **(B).** *B. subtilis* I2. Morphology of **(C).** *P. megaterium* H1 **(D).** *B. subtilis* I2 light-microscope photomicrograph showing the rod form

Supplementary Information

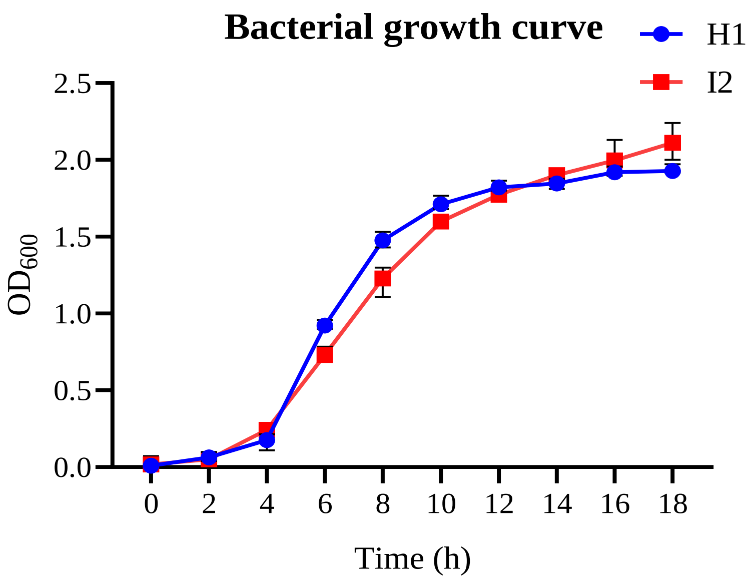

Figure S2. Antagonist bacteria growth curve in different intervals

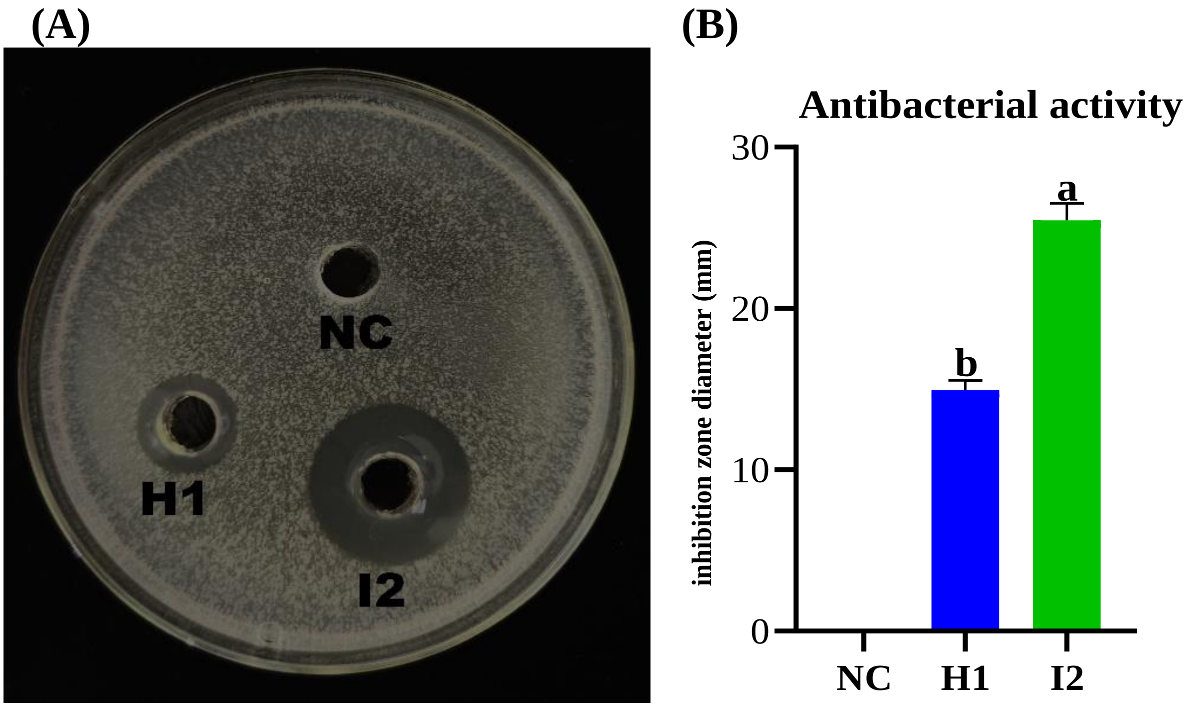

Figure S3. **(A):** Screening and inhibition of *E. amylovora* by antagonistic bacteria filtrate. **(B):** Diameter of inhibition zone of antagonist strains filtrate.
